# Neural correlates of ataxia severity in spinocerebellar ataxia type 3/Machado-Joseph disease

**DOI:** 10.1186/s40673-017-0065-7

**Published:** 2017-06-06

**Authors:** Carlos R. Hernandez-Castillo, Rosalinda Diaz, Aurelio Campos-Romo, Juan Fernandez-Ruiz

**Affiliations:** 1 CONACYT – Instituto de Neuroetología - Universidad Veracruzana, Xalapa, Ver Mexico; 2 Departamento de Fisiología, Facultad de Medicina, Universidad Nacional Autonoma de México, UNAM, CP 04510 Coyoacán, Ciudad de México México; 3 Unidad Periférica de Neurociencias, Facultad de Medicina, Universidad Nacional Autonoma de México UNAM, CP 04510 Coyoacán, Ciudad de México Mexico

**Keywords:** Atrophy, Machado-Joseph, SARA, Spinocerebellar ataxia, VBM

## Abstract

**Background:**

Spinocerebellar ataxia type 3/Machado-Joseph disease (SCA3/MJD) is an autosomal dominant inherited neurodegenerative disorder. Several post-mortem and imaging studies have shown cerebellar and brainstem atrophy. A number of studies have used volumetric regional information to investigate the relationship between neurodegeneration and the ataxia severity. However, regional analysis can obscure the specific location in which the degenerative process is affecting the brain tissue, which can be crucial for the development of new target treatments for this disease.

Here we explored the relationship between the gray matter degeneration and the ataxia severity on a cohort of SCA3 patients using a voxel-wise approach.

**Methods:**

Seventeen patients with molecular diagnose of SCA3 and 17 matched healthy controls participated in this study. Magnetic resonance imaging (MRI) brain images were acquired and voxel-based morphometry was used to obtain the grey matter volume of each participant. Ataxia severity in the patient group was evaluated using the scale for the assessment and rating of ataxia (SARA).

**Results:**

Group comparison revealed significant atrophy in SCA3 including bilateral cerebellum, vermis, brainstem, and occipital cortex. Significant negative correlations between gray matter volume and SARA scores were found in the cerebellum and the cingulate gyrus.

**Conclusions:**

These findings highlight the specific contribution of the cerebellum and the cingulate cortex to the ataxia deficits among the other regions showing neurodegeneration in SCA3 patients.

## Background

Spinocerebellar ataxia type 3/Machado-Joseph disease (SCA3/MJD) is an autosomal dominant inherited neurodegenerative disorder with a wide range of clinical manifestations [1]. SCA3/MJD is caused by an unstable CAG trinucleotide repeat expansion within the coding region of a gene located on chromosome 14q32.1 [2]. The predominant symptoms are progressive ataxia, ophthalmoplegia, spastic gait and peripheral neuropathy [1–3]. Previous morphometric SCA3/MJD MRI analysis revealed significant atrophy of the cerebellar hemispheres, cerebellar vermis, pontine base, middle cerebellar peduncle, medulla oblongata, cervical spinal cord and enlargement of the fourth ventricle [3–9]. However, only few studies have analyzed the possible relationship between brain degeneration and the ataxia severity in this disease [7–9]. In those reports, researchers have used manually predefined anatomical landmarks to segment and calculate the total volume of different anatomical structures [7], automated region segmentation to calculate cortical thickness and volume [8] or atlas predefined regions to later group them into one single region [9]. Those analyses led to broad correlations between volume of the cerebellum, brainstem and other cortical regions and the ataxia impairment.

However, a more accurate mapping of the disease-related degeneration and its association with the symptoms is key for the development of possible treatments/therapies for this specific type of patients. Here, we assessed the ataxia severity and gray matter degeneration in a cohort of SCA3/MJD patients by using the scale for the assessment and rating of ataxia (SARA) and whole brain voxel based-morphometry (VBM) to find voxel-wise associations between brain atrophy and motor impairment.

## Methods

### Participants

The patient group consisted of seventeen patients with a molecular diagnosis of SCA3/MJD (10 female; right-handed; mean age/SD, 40.1/11.9 years, for more detailed information look at Table 1). Motor impairment was measured using SARA [10], which has eight items, including tests of gait, stance, sitting, and speech, as well as a finger-chase test, finger-nose test, fast alternating movements, and heel-shin test. The control group consisted of 17 healthy volunteers that were age and gender matched. All participants gave written, informed consent before entering the study. The procedures carried out were in accordance with the ethical standards of the committees on human experimentation of the Universidad Nacional Autonoma de Mexico.

**Table 1:** Demographics of the patient group

ID	Gender	Age	Age at onset	SARA
P01	F	49	39	26
P02	M	58	47	19.5
P03	F	45	25	26
P04	M	37	32	12.5
P05	F	35	33	6.5
P06	F	23	20	6
P07	F	42	35	12
P08	F	24	20	9
P09	F	18	16	2.5
P10	F	45	39	8
P11	M	43	40	14.5
P12	M	34	29	18
P13	M	59	49	19.5
P14	M	33	28	9.5
P15	F	40	30	20
P16	M	56	46	14.5
P17	F	46	41	1.5

### Image acquisition

All images were acquired using a 32-channel quadrature head coil in a 3.0-T Achieva MRI scanner (Phillips Medical Systems, Eindhoven, The Netherlands). Foam-rubber cushion was used for fixing the head of the subject in place, so as to minimize any head movements. The high-resolution anatomical acquisition consisted of a 3-D T1 Fast Field-Echo sequence, with TR/TE of 8/3.7 ms, FOV of 256 × 256 mm, flip angle 25° and an acquisition and reconstruction matrix of 256 × 256, resulting in an isometric resolution of 1 × 1 × 1 mm.

### Voxel-based morphometry

Gray matter volume (GMV) measurements were performed using voxel based morphometry (VBM) as implemented on FSL (FMRIB, Oxford, UK) following the standard procedure as previously reported [11]. Using the FSL randomise tool, a two-sample t test was performed between the SCA3 group and controls. Significance was defined as *p <* 0.05 after correcting for multiple comparisons using the randomized permutation method (TFCE). For the SCA3 group, whole-brain correlation maps were created by calculating the Pearson’s partial correlation between the GMV and SARA scores including age in the analysis. Partial correlation maps were corrected for multiple comparisons by using false discovery rate (FDR) with a *p* value < 0.05.

## Results

**Fig. 1:**
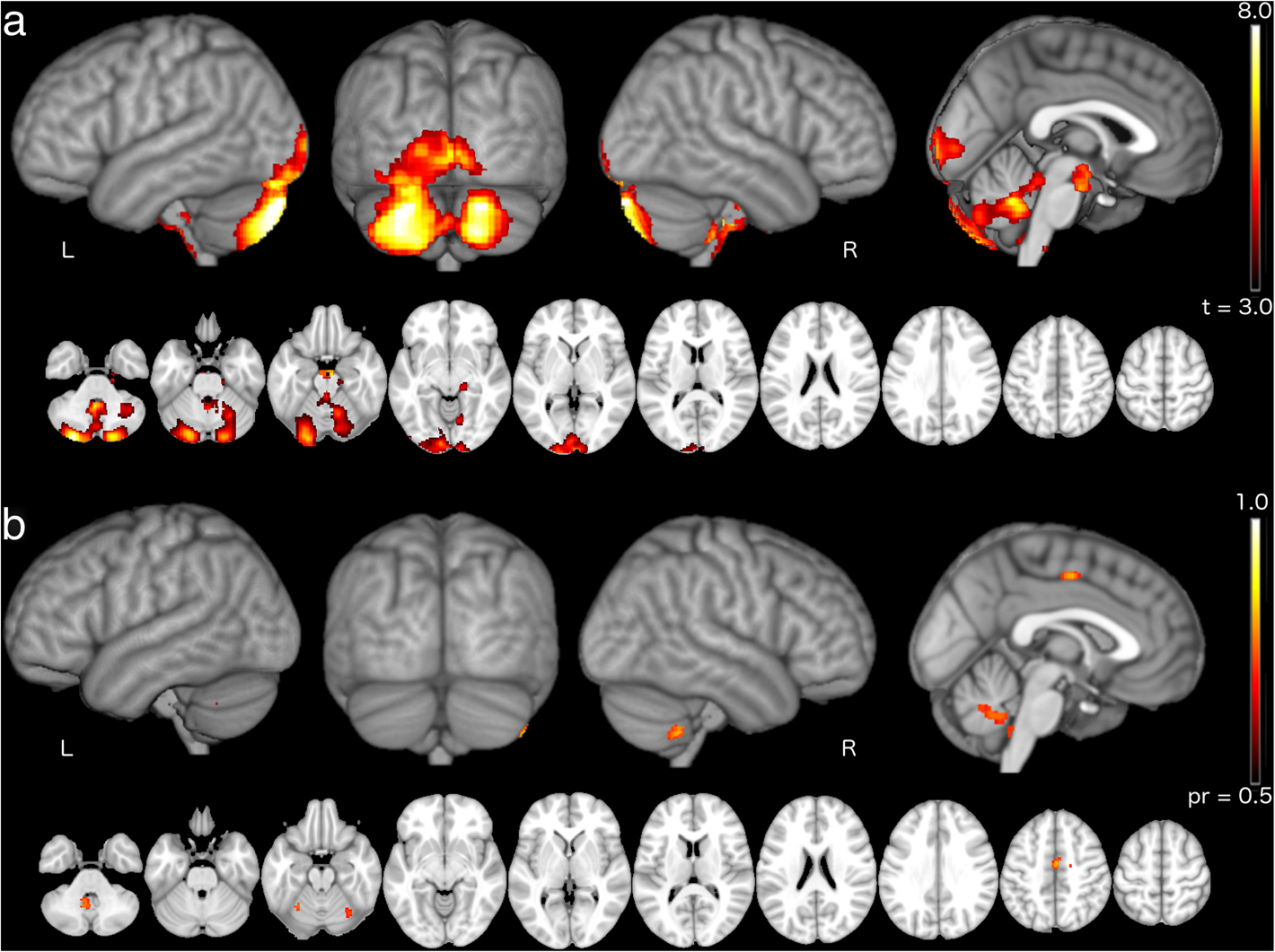
Brain regions showing gray matter atrophy and SARA-GMV correlation. **a** Significant gray matter atrophy in patients compared with controls; **b** significant partial correlations between patients’ GMV and SARA

VBM analysis revealed gray matter atrophy in SCA3 patients compared to the control group (Fig. 1a) involving bilateral cerebellum, vermis, brainstem and the occipital cortex (Table 2). Four significant negative correlations were found between GMV and SARA scores in the SCA3 group (Fig. 1b) in regions of the cerebellum and the cingulate gyrus (Table 3).

**Table 2:** Brain regions showing significant gray matter degeneration in SCA3/MJD patients

Anatomical region	X	Y	Z	Cluster	t
Left posterior cerebellum Crus II	−26	−86	−36	2633	7.73
Right posterior cerebellum Crus II	20	−88	−32	1419	7.31
Right brainstem	4	−14	−22	224	6.59
Vermis IX	−2	−54	−34	114	6.45
Pons	2	−14	−24	219	6.19
Right anterior cerebellum lobule V	12	−58	−20	160	5.1
Left lingual gyrus BA17	−12	−100	−4	132	4.42
Left anterior cerebellum lobule I-IV	−8	−46	−22	213	3.59

Coordinates in MNI space in mm. BA = Brodmann Area

**Table 3:** Brain regions showing significant correlation between GMV and SARA

Anatomical region	X	Y	Z	Cluster	PC
Right cerebellum declive lobule VI	32	−64	−14	191	−0.825
Left cerebellum culmen lobule VI	−28	−50	−18	180	−0.816
Left cerebellum tonsil lobule IX	−8	−58	−38	155	−0.792
Right paracentral lobule BA6	4	−8	48	132	−0.779

Coordinates in MNI space in mm. BA = Brodmann Area, PC = partial correlation

## Discussion

In this study, we analyzed the relationship between gray matter loss and SARA scores in SCA3 patients. As expected, significant negative correlations between SARA scores and GMV were found in the cerebellum, but also in the cingulate cortex.

Previous reports have shown the close relationship between the extent of the brain atrophy, predominately in the cerebellum, and a variety of symptoms in different SCA subtypes [7–9, 12, 13]. Accordingly, our analysis showed a significant negative correlation between GMV and the SARA score in the bilateral lobule VI, extending to lobule V, which are involved in sensorimotor processing as suggested by deficits in stroke patients [14, 15]. Lobule IX, which its GMV also correlated with SARA, is not only considered essential for visual guidance of movement [14], but its damage has been related to gait and balance impairment [15].

The only extra-cerebellar region where the GMV correlated with SARA score was the dorsal anterior cingulate, known to be critically involved in motor functions [16]. A previous report showed a significant degeneration in SCA3 in this area [6], however, our analysis also showed a negative correlation between GMV and the ataxia score, corroborating the functional relevance of this deterioration as shown by patients with lesions in this area, whom often show deficits in spontaneous initiation of movement and speech, as well as inability to suppress externally triggered motor subroutines [16].

## Conclusions

In Conclusion, we report specific key areas where the GMV shows a close relationship with the ataxia impairment in SCA3. These findings add to previous reports [7, 9], while providing a more accurate localization of the SCA3/MJD ataxia-related areas.

## Abbreviations

FDR: False Discovery Rate
GMV: Gray Matter Volume
MDJ: Machado-Joseph Disease
MRI: Magnetic Resonance Imaging
SARA: Scale for Assessing and Rating of Ataxia
SCA3: Spinocerebellar Ataxia 3
VBM: voxel-Based Morphometry
